# The Prion-Like Spreading of Alpha-Synuclein in Parkinson’s Disease: Update on Models and Hypotheses

**DOI:** 10.3390/ijms22158338

**Published:** 2021-08-03

**Authors:** Asad Jan, Nádia Pereira Gonçalves, Christian Bjerggaard Vaegter, Poul Henning Jensen, Nelson Ferreira

**Affiliations:** 1 Danish Research Institute of Translational Neuroscience (DANDRITE), Nordic EMBL Partnership for Molecular Medicine, Department of Biomedicine, Aarhus University, 8000 Aarhus, Denmark; npg@dandrite.au.dk (N.P.G.); cv@biomed.au.dk (C.B.V.); phj@biomed.au.dk (P.H.J.); 2 International Diabetic Neuropathy Consortium (IDNC), Aarhus University Hospital, 8200 Aarhus, Denmark

**Keywords:** Parkinson’s disease, alpha-synuclein, prion-like, neurodegeneration

## Abstract

The pathological aggregation of the presynaptic protein α-synuclein (α-syn) and propagation through synaptically coupled neuroanatomical tracts is increasingly thought to underlie the pathophysiological progression of Parkinson’s disease (PD) and related synucleinopathies. Although the precise molecular mechanisms responsible for the spreading of pathological α-syn accumulation in the CNS are not fully understood, growing evidence suggests that de novo α-syn misfolding and/or neuronal internalization of aggregated α-syn facilitates conformational templating of endogenous α-syn monomers in a mechanism reminiscent of prions. A refined understanding of the biochemical and cellular factors mediating the pathological neuron-to-neuron propagation of misfolded α-syn will potentially elucidate the etiology of PD and unravel novel targets for therapeutic intervention. Here, we discuss recent developments on the hypothesis regarding trans-synaptic propagation of α-syn pathology in the context of neuronal vulnerability and highlight the potential utility of novel experimental models of synucleinopathies.

## 1. Introduction

Parkinson’s disease (PD) is a major neurodegenerative disease causing progressive motor disability in individuals over 55–60 years of age and affects both genders with a slight male preponderance [1,2,3]. Clinical PD is defined by the cardinal signs of TRAP (resting Tremor, Rigidity, Akinesia/bradykinesia and Postural/gait instability) [4], which respond to L-dopa therapy (especially tremor and rigidity) [1,2]. The etiology of PD-like motor disability, termed parkinsonism, is predominantly of idiopathic nature, but can also be observed in other neurological conditions (e.g., post-encephalitis, repetitive traumatic brain injury, progressive supranuclear palsy) [5]. Moreover, the clinical presentation of idiopathic PD can also be heterogeneous and is further sub-classified into additional variants (e.g., tremor dominant, akinetic-rigid, early disease onset or mixed) with distinct responses to the existing therapeutic modalities [6,7,8]. Lastly, long-standing PD invariably results in cognitive decline and dementia in as many as 30–40% of the cases, especially in patients with the late-onset disease [1,2]. However, it is becoming increasingly evident that PD has a long (15–20 years) prodromal phase during which the affected individuals experience non-motor symptoms, particularly anosmia, autonomic dysfunction, constipation and REM sleep behavior disorder (RBD) [9,10]. In the backdrop of clinical scenarios, the neuropathological diagnosis of PD requires two features: (i) depigmentation/demelanization of the substantia nigra-pars compacta (SNpc) due to the pathological loss of dopaminergic neurons and (ii) the presence of Lewy related α-synuclein (α-syn; gene symbol *SNCA/PARK1*) pathology (LRP), e.g., Lewy bodies (LBs) and Lewy neurites (LNs), across several brain regions, primarily in the brainstem nuclei [1,11,12]. A detailed overview of PD neuropathology is beyond the scope of this review and can be accessed elsewhere [5,11]. However, it suffices to mention that aggregation and deposition of α-syn in the CNS, either due to genetic predisposition and/or the presence of factors in the local microenvironment that are conducive to α-syn aggregation (i.e., impaired redox homeostasis, ionic imbalance and neuroinflammation [13,14,15]), contribute to the proteopathic stress with detrimental consequences for the neuronal function and/or survival [16,17]. Hence, currently prevailing consensus maintains that PD is the result of chronical loss of dopaminergic neurons in SNpc, which culminates with dysregulated modulatory innervations into the striatum and resultant dysfunction of the nigro-striatal-cortical circuitry in the basal ganglia [1,2,11]. However, post-mortem studies in pathologically diagnosed PD show that a variable degree of LRP is also found in several extra-nigral locations (nuclei) in the brainstem, including the dorsal motor nucleus of vagus-dmX and intermediate reticular zone in the caudal brainstem, and more rostrally in the gigantocellular reticular nucleus (GRN), locus coeruleus (LC) and subcoeruleus complex, raphe nuclei, the tractus solitarius, SNpc, the pedunculopontine nucleus-PPN and the ventral tegmental area [18,19]. In late-stage PD, localized LRP has also been detected in distinct nuclei of the basal forebrain, thalamus, hypothalamus, the olfactory and basolateral portions of the amygdala, the anterior olfactory nucleus, CA2 of the hippocampus, as well as the insular, cingulate and prefrontal cortices [11,20]. These observations suggest that, although not unique to PD, the distribution of LRP in PD is not random and exhibits a predilection for distinct neuronal populations and their connectivity [11,18,21,22]. Accordingly, recent opinions have sought to reconcile the symptomatology of PD with the known aspects of α-syn LRP, especially the pattern(s) of LRP initiation and propagation in the nervous system and the consequent dysfunction in affected neuronal populations [9,20,23].

## 2. α-Syn Aggregation and Cytotoxicity

α-Syn is a 140-amino-acid cytoplasmic protein that is mostly found within presynaptic nerve terminals and is involved in the assembly of the SNARE complexes [24,25]. α-Syn contains an amphipathic lysine-rich N-terminal region, which plays a crucial role in lipid-binding [26], and an acidic carboxyl-terminal region, which is enriched with acid residues and that has been implicated in the protein’s chaperone-like activity [27]. The central domain of α-syn is known as the non-amyloid-component (NAC) (61–95) and contains a highly hydrophobic motif, essential for α-syn aggregation [28]. Burgeoning genetic and neuropathological evidence suggests that the abnormal aggregation of monomeric α-syn into intracellular insoluble protein inclusions in the brain (e.g., LBs and LNs) plays a key role in the development of several adult-onset neurodegenerative diseases, including PD, Multiple System Atrophy (MSA) and Dementia with Lewy Bodies (DLB), all collectively known as synucleinopathies. LBs are spherical cytoplasmic inclusions, which are 5–25 µm in diameter, and mainly composed of aggregated α-syn, with a dense eosinophilic core surrounded by aggregated α-syn brighter halo [29]. Several point mutations (A30P, E46K, H50Q, G51D or A53T) within the *SNCA*, as well as duplication and triplication of *SNCA*, are closely associated with rare early-onset familial PD and DLB cases, demonstrating an unequivocal connection between α-syn and neurological disease [30,31,32,33]. Nevertheless, with the exception of the aforementioned rare familial forms, the great majority of PD cases are sporadic, suggesting that PD etiology is most likely multifactorial, involving a complex interplay between aging, genetic susceptibility and environmental factors [1]. Therefore, understanding the earliest physiological to pathological events underlying α-syn misfolding and abnormal aggregation is of utmost importance, since they provide opportunities for therapeutical intervention.

Despite recent advances, the nature of precise native conformation of α-syn under physiological conditions remains elusive. However, it is widely accepted that α-syn primarily occurs as an intrinsically disordered monomer in the cytosol [34], with few tertiary interactions between the C-terminus and the central hydrophobic NAC region and the N-terminus of the protein [35,36]. A wide variety of conditions have been found to induce α-syn misfolding and aggregation in vitro, including acidic pH [37,38], increased temperature [37], molecular crowding [39], divalent and trivalent metal ions such as aluminum, copper(II), iron(III), cobalt(III) and manganese(II) [40], organic solvents [41], lipids with high solubility in aqueous solution and short hydrocarbon chains [42], heparin and other glycosaminoglycans [43], polycations [44], pesticides [45] and α-syn binding proteins [46,47,48]. In addition, α-syn can undergo extensive post-–translational modifications (PTMs) that are known to modulate its neurotoxicity and its propensity to aggregate, including phosphorylation [49,50,51,52], ubiquitination [53,54], nitration [55,56], sumoylation [57,58], truncation [59,60] and N–terminal acetylation [61,62]. Among the PTMs, approximately 90% of the α-syn aggregates present in LBs are phosphorylated on the serine residue-129 (p-S129), hence S129 hyperphosphorylation of α-syn has been widely regarded as a pathological hallmark of PD and related synucleinopathies [63]. Whether the disease-associated α-syn phosphorylation stimulates or hampers α-syn aggregation, and its neurotoxicity in a pathophysiological context, remain debatable [64,65]. Resembling other aggregation-prone proteins, α-syn self-assembly exhibits a sigmoidal profile in biochemical assays measuring protein aggregation [66,67,68,69,70,71]. The formation of α-syn fibrils typically follows a nucleation-dependent mechanism, consisting of an initial lag phase, followed by a growth phase of elongation and a plateau phase of fibril maturation [72,73]. The oligomeric aggregates can be structurally categorized according to their size and shape, and functionally classified as on-pathway and off-pathway, depending on whether they evolve to form mature amyloid fibrils or, alternatively, result in amorphous, non-fibrillar assemblies [74,75]. Moreover, whether low molecular weight α-syn oligomers, rather than mature fibrils, are the most toxic entities underlying α-syn toxicity remains uncertain. In this regard, a robust dopaminergic loss in the substantia nigra in transgenic animals expressing α-syn variants that form ring/pore-like oligomers has been reported (i.e., E57K and E35K), whereas the α-syn variants that rapidly form fibrils were found to be comparatively less toxic in these experiments [76]. Of note, this study was based on an ectopic lentivirus expressing system, thus, partly limited by the fact that α-syn is overexpressed locally. It is plausible that the fibril forming variants can be actively recruited by LBs into aggresome-like structures, and prevent their abnormal interactions with other cytoplasmic proteins and deleterious effects on the function of cellular organelles [77,78]. A supporting role of oligomeric α-syn neurotoxicity is also suggested by immunotherapy using antibodies targeting oligomeric α-syn, which rescued motor dysfunction in a PD transgenic mouse model [79]. In line with these observations, increased levels of soluble α-syn oligomers have been detected in brain and cerebrospinal fluid (CSF) of patients with LB pathology compared to healthy age-matched controls [80,81,82,83].

Compounding evidence in animal models and cell cultures, including neuronal cultures, implicate a pathogenic role of pathological α-syn aggregation in triggering detrimental effects on the synaptic function, purportedly via calcium dyshomeostasis, mitochondrial impairment, endoplasmic reticulum (ER) stress, defective autophagy, neuroinflammation and oxidative stress [15,17,84]. It has also been suggested that α-syn aggregation in the presynaptic terminals and sequestration into the inclusions affects the assembly of SNARE complexes, thus decreasing the efficiency of dopamine release [85]. Moreover, several synaptic proteins and neurotransmitter receptors (e.g., NMDA glutamate receptors) have been identified as putative interaction partners of α-syn, and these aspects have been reviewed elsewhere [86,87].

## 3. α-Syn Cell-to-Cell Propagation

Prions are infectious agents in which the conformationally altered protein PrP^Sc^ recruits and corrupts its normal counterpart PrP^C^ generating self-propagating misfolded species, which can spread from cell-to-cell [88]. In recent years, it has been demonstrated that several amyloid-forming proteins possibly share an analogous prion-like spreading mechanism, including α-syn [89,90], β-amyloid [91,92], tau [93,94] and Huntingtin [95,96]. Accordingly to the Braak model, PD neuropathological staging follows a highly stereotypical and spatiotemporal progression for the Lewy pathology, suggesting propagation of misfolded α-syn through vulnerable neuroanatomically connected pathways (elaborated below under Section 4) [18]. Initial evidence supporting a prion-like propagation of α-syn came from the observation of α-syn aggregation in grafted fetal mesencephalic progenitor neurons several years after transplantation, and implied host-to-graft Lewy pathology transmission [97,98]. Since then, accumulating evidence has shown that α-syn seeds formed from recombinant proteins or aggregate-containing lysates from diseased brains can propagate following the prion-like paradigm in neuronal cells, in organotypic slice cultures and in rodent models of PD [48,89,90,99,100,101,102,103,104,105,106].

Similar to PrP^Sc^, α-syn can self-assemble into β-sheet-rich amyloid fibrils giving rise to epidemiologically and histopathologically distinct neurodegenerative diseases. In PD and DLB, widespread α-syn aggregation is observed not only in α-syn-expressing neuronal populations (such as LBs and LNs) but also in neighbouring astroglial cells [107,108]. In comparison, the neuropathology of MSA is principally characterized by α-syn aggregates in the oligodendroglia as glial cytoplasmic inclusions (GCIs) and in instances of neuronal nuclear inclusions in discrete brain regions (e.g., base of pons) [12]. In addition, α-syn pathology is also observed in Alzheimer’s disease (AD) [109,110], and in about 20% of neurologically normal elderly individuals as the incidental LB disease (iLBD) [111].

The detailed molecular pathways underlying α-syn exocytosis, the potential interaction of α-syn with extracellular components and the conformational properties of released α-syn in terms of aggregation state remain largely elusive. Misfolded α-syn seeds are secreted from donor cells to the interstitial compartment as naked protein or in vesicles (e.g., exosomes) [112,113,114,115,116,117]. Although the initial focus of this mechanistic research was primarily in neurons, compounding evidence has shown that microglial exosomes may significantly contribute to the progression of α-syn pathology, and potentially serve as a therapeutic target for PD [118,119]. Once in the extracellular space, α-syn seeds have been shown to be taken up by neighbouring cells in culture via several routes, including direct penetration of the plasma membrane, fluid-phase or receptor-mediated endocytosis (e.g, via lymphocyte-activation gene 3 in cultures of primary neurons) or fusion of plasma-exosomal membranes [112,113,114,120,121]. In addition to the release and internalisation mechanisms, tunnelling nanotubes that directly connect two adjacent cells have also been reported to play a role in cell-to-cell transfer of pathological α-syn assemblies [122,123]. Once inside the recipient cell, α-syn seeds purportedly undergo multiple PTMs (e.g., truncation, phosphorylation, ubiquitination), that facilitate interactions with endogenous α-syn monomers and other cytosolic proteins, and further promote α-syn aggregation and propagation [112,113,114]. Therefore, potential therapeutic approaches to modulate these processes include antibodies that specifically target the α-syn seeds or the cellular release and uptake machinery.

Lastly, the involvement of α-syn aggregates in several diseases that exhibit dissimilar phenotypic traits, together with the fact that synthetic α-syn monomers can form polymorphs with distinct conformations and biological activities [124,125,126,127], has led to the recent hypothesis that multiple α-syn strains may underlie the clinical heterogeneity observed in synucleinopathies and other neurodegenerative diseases [48,128,129,130]. Indeed, α-syn inclusions isolated from MSA brains have unique ultrastructural features that differ from those of individuals with DLB [131,132]. Interestingly, it has been shown that oligodendrocytes, but not neurons, phenoconvert LB-like α-syn fibrils into a GCI-like strain, highlighting the fact that the oligodendroglial intracellular milieu determines how MSA-associated α-syn strains are generated [130]. Supporting these findings, it has been found that sub-stoichiometric concentrations of oligodendroglial protein p25α redirects α-syn aggregation into a unique α-syn/p25α strain with a different structure and enhanced in vivo neurodegenerative properties [48]. Taken together, these observations highlight the importance of both misfolded seeds and intracellular milieu in the formation of α-syn strains.

## 4. α-Syn Propagation in the Clinical Pathology of PD: Models and Hypotheses

Although a single unifying hypothesis is still lacking, the prevailing viewpoints on the pathogenic role of α-syn aggregation in PD mainly posit the following set of arguments: (**i**) pathological α-syn aggregation is triggered in extra-nigral location(s), purportedly in contact with the external environment, and subsequently propagates in the nervous system following neural connectivity [18,133]; and (**ii**) the profound neurodegeneration of neuronal populations (primarily SNpc) in PD reflects selective vulnerability to pathogenic processes, including α-syn induced proteopathic stress [20,134]. Despite some differences, largely due to the basis of the primary evidence, advances in investigative methodology such as brain imaging and refinements in the experimental models will possibly reveal some degree of overlap in these viewpoints. In the following sections, we will summarize the salient features of these hypotheses and provide some perspectives on the significance of extra-nigral α-syn LRP for PD.

### 4.1. ‘Dual-Hit’ Hypothesis

According to this hypothesis, pathological α-syn aggregation is initiated in peripheral (i.e., extra-cerebral) locations such as olfactory epithelium and/or gut mucosa in response to the exposure to environmental factors, presumably a neurotropic viral pathogen or a toxin [135]. Based on post-mortem neuropathological assessment of LRP in PD and iLBD, Braak and colleagues proposed that the α-syn LRP in PD develops in defined spatiotemporal patterns (stages) and is possibly multifocal in origin. Briefly, in the earliest phase (stage 1), LRP is detected in the dmX/IX and within few projections of the medullary intermediate reticular zone, peripheral autonomic ganglia and spinal cord (and anterior olfactory nucleus). Subsequently, there is a caudo-rostral propagation into the pontine tegmentum (stage 2; GRN, LC and raphe magnus), midbrain (stage 3; SNpc), basal forebrain and olfactory areas (stage 4) and eventually neocortical regions (stages 5–6) [18,133]. Given the observations that symptomatic PD usually indicates the loss of 30–70% of SNpc dopaminergic neurons in the ventrolateral tier of SNpc (and their striatal terminals) [136,137,138], i.e., stage 3 of Braak scheme, this neuropathological scheme in its simplest form arguably serves to categorize PD as presymptomatic (Braak stages 1–2), early symptomatic (Braak stages 3–4) and late symptomatic (Braak stages 5–6) phases [20]. It is noteworthy that not all PD cases, between 17% and 47%, show clinical correlation with the distribution of LRP following this scheme [8,20,139]. For instance, in some studies there was a remarkable lack of LRP in dmX despite significant involvement of higher brainstem or cortical regions in up to 8% of the examined specimen [140]. Nevertheless, these data show that pathological α-syn accumulation in the form of LRP preferentially affects distinct neuronal populations/nuclei and that neuronal susceptibility to LRP accumulation is possibly modulated by genetic factors [20,141]. Furthermore, non-physiological α-syn deposition has also been reported in peripheral sites, presumably in the areas of innervations of peripheral autonomic nerves [134,142].

**Figure 1:**
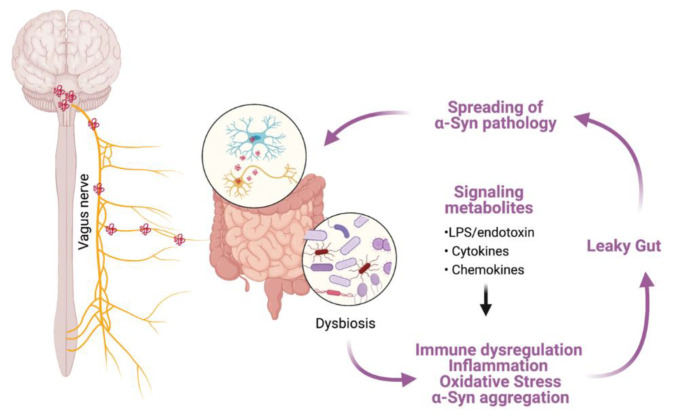
A schematic representation of hypothesized α-syn aggregation and spreading from the ENS towards the CNS via vagus nerve. Environmental factors, including changes in the gut microbiota (dysbiosis), are hypothesized to initiate pathological processes within the enteric nerve cell plexus, provoking mucosal inflammation and oxidative stress and thereby inducing abnormal aggregation of α-syn. Increased permeability of the intestinal barrier (‘leaky gut’) will ultimately provide a route of transmission for the ENS-formed α-syn seeds into the brain. Structures are not drawn to scale. The illustration was created in biorender.com (accessed on 3 August 2021).

In this mechanistic model of caudo-rostral α-syn neuroinvasion, the enteric nervous system (ENS) of the gastrointestinal tract (GIT) and the associated autonomic ganglia have been proposed as the putative sites of origin for α-syn aggregation in the periphery [133]. α-Syn aggregation induced dysfunction in the ENS has, in turn, been implicated in several GIT related non-motor symptoms in PD (i.e., constipation) [143]. The neuropathological findings that support the role of ‘gut-brain’ axis in PD have recently been discussed in several review opinions [144,145,146] and some key aspects of this model are presented below. Within the ENS, α-syn immunopositivity has been reported in the intramuscular myenteric and submucosal Meissner’s plexuses in the gastric, duodenal and colonic biopsies during the prodromal stages of PD (i.e., in the absence of motor disability) [18,147,148], as well as in patients with idiopathic rREM sleep behaviour disorder (iRBD), which is present in as much as one-third of PD patients and considered a strong indicator of prodromal PD [149]. In population-based studies, truncal vagotomy (i.e., division of the anterior and posterior trunks proximal to the gastro-esophageal junction; used mainly in the treatment of complicated peptic ulcer disease) has been shown to reduce the risk of developing PD by 40–50% after 5 years post-procedure [144,150,151]. Furthermore, positron emission tomography (PET) studies using [^11^C]-donepzil, a surrogate marker for assessing cholinergic parasympathetic gut innervation, show reduced signal in the colon and small intestine during early-stage PD [152] and in the cohorts of subjects with iRBD [153]. These observations reinforce the idea that, in a subset of PD cases, initial α-syn pathology may originate within the ENS and subsequently involve autonomic ganglia peripherally and dmX centrally. In support of this viewpoint, exogenous inoculation of PD brain lysates or preformed fibrillar (PFF) α-syn into the GIT of wild type rats was followed by the emergence of α-syn accumulation in ENS and subsequently in the dmX as early as 2–3 days post-inoculation [154]. Similarly, PFF α-syn inoculation in the gastric or intestinal wall of C57BL/6 mice resulted in substantial α-syn phosphorylation (p-S129) in the dmX, which was prevented by vagotomy prior to the PFF α-syn inoculation [146,155]. Although, the spreading of α-syn inclusions beyond the dmX has not been consistently observed in these experiments, their findings provide support for the notion that α-syn aggregation in ENS can spread centrally into the CNS, and mimics a pattern observed following ectopic induction of α-syn expression in the vagus nerve of rodents using recombinant adeno-associated viruses (rAAV). With regards to the factors that promote de novo α-syn in the ENS, several mechanisms have been implicated including the altered composition of gut microbiome in PD [156], chronic helicobacter pylori infection [157], disruption of intestinal epithelial barrier due to bacterial and environmental toxins leading to a ‘leaky gut’ [158,159] and potentially a pro-inflammatory milieu due to dysregulated immune response in the GIT [160,161] (Figure 1).

However, it is worth mentioning that none of the neuropathological studies published to date have identified isolated α-syn accumulation that is localized only to the gut component of ENS and, in the absence of intracerebral LRP, that eventually progressed into clinical PD [145]. Even if such cases of localized α-syn pathology in ENS do exist, their detection is a highly challenging task technically as the human GIT is 8–10 m long and rigorous analyses would necessitate a large number of tissue sections. Furthermore, some studies have also shown instances of α-syn immunodetection in the intestinal biopsy specimen from neurologically normal individuals [162,163]. As in the case of intracerebral LRP in iLBD [164], it remains unsettled if these individuals eventually will progress to develop a Lewy body disorder or represent a population with an intact ENS function (as revealed by the expression of ENS neurotransmitter molecules [163] that potentially compensates in response to local α-syn aggregation.

Based on these findings, a significant development in the field has been to investigate if α-syn misfolding and propagation in the nervous system occurs in a ‘prion-like’ fashion, i.e., templating of endogenous physiological α-syn into misfolded conformers formed in situ or received from a neuroanatomically connected location [23,165]. Due to the absence of validated biomarkers that can be used in longitudinal studies and the absence of clinical assessment that could reveal PD progression from non/early-symptomatic to symptomatic stage, unequivocally demonstrating a causative role of LRP propagation with the disease stage is certainly a daunting task [8,20]. Pivotal evidence in PD brain was provided by the histological assessment of heterologous fetal transplants in striatum, which revealed that some of the neurons developed LRP after approximately a decade [97,98]. The implication that these observations indicate bona fide host-to-graft propagation has been contested [134]. For instance, even at late stage PD, LRP in medulla remains largely confined within specific cell populations and does not invade neighboring neurons, e.g., LRP is usually found in dmX, intermediate reticular zone and raphe magnus, while the neighboring nuclei are spared [166]. However, these findings emphasize the role of pre-existing LRP in altering local microenvironment conducive to de novo LRP formation and/or progression. In this regard, several studies in cellular and animal models support such ‘prionoid’ behavior of α-syn, i.e., trans-synaptic propagation and templating, although fully recapitulating the spectrum of PD associated LRP combined with preferential loss of SNpc dopaminergic neurons has been challenging [23,167,168,169]. Despite some limitations, these animal models have been instrumental in elucidating that in vivo inoculation with exogenous fibrillar α-syn, LRP containing human brain extracts or the overexpression of α-syn via viral mediated somatic gene transfer induces the aggregation of endogenous α-syn in recipient neurons (and glial cells) [23,90,101,170,171,172,173]. Such de novo induced aggregated α-syn inclusions, in most instances, also contain markers of PD associated LRP, including α-syn phosphorylation at serine residue 129 (p-S129) along with co-detection of ubiquitin and/or the general inclusion marker p62 (sequestosome) protein [23,169]. Importantly, in animal studies several groups have shown that intracerebral or peripherally induced α-syn aggregation (including direct nerve injections) spreads into connected neuronatomical tracts and the aggregation is also not random [101,102,174,175]. Nevertheless, widespread, but circumscribed, LRP-like α-syn deposition in the CNS seems to be more pronouncedly observed in models using fibrillar α-syn and less so using oligomeric α-syn or virally induced α-syn overexpression models [20,23,169,171]. Overall, existing evidence suggests that, in these experimental models, the initial phase of (arguably) trans-synaptic pathological α-syn spreading occurs in a retrograde fashion, although the identification of factors mediating and/or promoting α-syn cell-to-cell transfer remains an evolving field [23].

### 4.2. Selective Neuronal Vulnerability/Threshold Hypothesis

**Figure 2:**
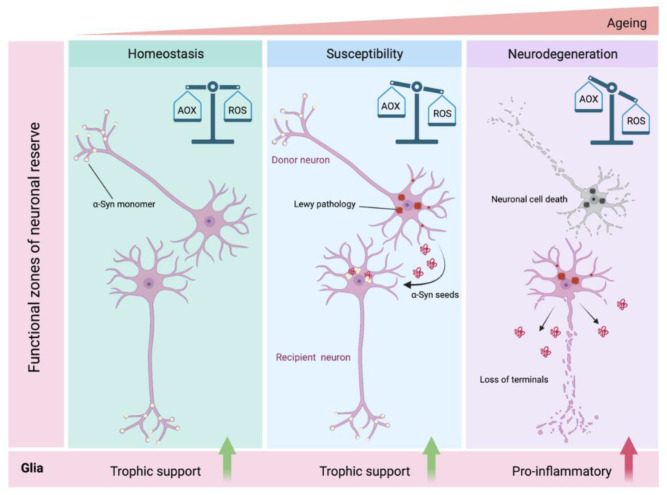
Schematic depiction of hypothesized α-syn neuron-to-neuron transmission and intracellular redox imbalance resulting in neurodegeneration. Under normal homeostatic conditions, neuronal α-syn exists in soluble non-aggregated conformations and the anti-oxidant (AOX) scavenging mechanisms are at equilibrium with intracellular reactive oxygen species (ROS) generation. Misfolded α-syn perturbs cellular redox balance in favor of excessive ROS, which is further aggravated by additional susceptibility/risk factors (e.g., genetic risk factors and ageing) that promote pathological α-syn aggregation and proteopathic stress [1,2]. Subsequently, cell-to-cell transmission of α-syn seeds from the affected neurons (depicted as donor neuron) via the neuroanatomical projections onto additional neuronal populations results in transmission of α-syn pathology into the recipient neurons. In the receiving neuron, the newly internalized seeds recruit endogenous soluble α-syn and further template a vicious cycle of α-syn aggregation and neurotoxicity. In established (i.e., long-term) α-syn neuronal pathology, there is profound dysregulation of AOX/ROS balance which is associated with loss of synaptic terminals and neuronal demise. The neuroglial cells modulate these processes by providing trophic support (e.g., glia derived neurotrophic factor- GDNF) which serves to maintain pro-survival local microenvironment [185,186,187,188,189]. However, relentless disease progression and ensuing neurodegeneration are strong triggers for neuroinflammatory response. Structures are not drawn to scale. The illustration was created in biorender.com (accessed on 3 August 2021).

The basic tenet of this viewpoint is that certain neuronal populations, due to their inherent cellular and network properties, are more vulnerable to α-syn induced proteopathic stress and susceptible to changes in the local microenvironment (e.g., neuroinflammation, metabolic deficits) [20,134,176]. As suggested earlier, the pathogenic roles of misfolded α-syn on mitochondrial function, autophagic flux and altered ion balance (e.g., calcium) are very well studied in cell-based paradigms and have been supported by findings in animal models of synucleinopathies [84,177]. Current opinion suggests that the neurons typically affected by pathological α-syn accumulation are CNS projection neurons with thin, long unmyelinated or poorly myelinated axons, and with comparatively higher axonal terminals (i.e., hyperbranching axons) [138,178]. In this regard, it has been estimated that a single dopaminergic neuron makes up to a quarter million striatal synapses in the rat brain, while in human brain the number of vesicle release sites can be 10-fold higher [20,179]. Several studies show that the neurodegenerative changes initially comprise the loss of terminals, with subsequent swelling of neuronal soma and some degree of neuronophagia by microglial cells [180,181,182]. In addition, the extensive hyperbranching places extra burden on metabolic regulation, (e.g., to meet the demand for axonal transport) and compromises scavenging capacity to mitigate oxidative stress (highlighted in Figure 2) [183,184,185].

Supporting this notion of impaired redox homeostasis, several immunohistochemical studies have shown aberrant localization of redox regulating molecules in association with LRP in PD SN, including nuclear factor erythroid 2-related factor 2 (NRF2/Nrf2) [186,187], NRF2 inhibitor Kelch-like ECH-associated protein 1 (Keap1) [188], anti-oxidant heme oxygenase (HO-1) [189] and anti-xenobiotic NAD(P)H quinone dehydrogenase 1 (NQO1) [190]. Moreover, the neuronal populations containing LRP in PD belong to diverse neurotransmitter systems (dopamine, serotonin, noradrenaline and acetylcholine) [23], yet not all neurons containing pathological α-syn inclusions show relentless neurodegeneration (e.g., tuberomamillary nucleus in hypothalamus [191]) as observed in the SNpc.

Moreover, in SNpc, the number of LRP/total α-syn immunopositive neurons does not correlate with disease severity and is stable over time, with ~3.6% of the neurons affected on average [192,193]. In contrast, dopamine transporter (DAT) density is reported to be inversely correlated to the total α-syn in SN than LRP burden, arguably favoring defective axonal transport [194,195]. Conversely, some brain regions exhibit variable loss of neurons (e.g., supraoptic nucleus) in the relative absence of LRP [191]. Apart from these structural/cytoarchitectural features discussed above, certain functional properties have also been posited as factors that predispose neurons to α-syn induced neurotoxicity (i.e., the threshold hypothesis) [134], which do not necessarily correlate with LRP burden in PD. Electrophysiological measurements show that the SNpc dopaminergic neurons possess slow, tonic and autonomic pacemaking activity characterized by broad spikes [196,197,198]. These neurons also exhibit a sustained opening of calcium cav1 channels, with large intracellular Ca^2+^ oscillations and low intrinsic Ca^2+^ buffering [196,199,200,201]. The neighboring dopaminergic neurons in VTA, which are less susceptible to neurodegeneration, also exhibit autonomic pacemaking and broad spikes, but possess comparatively smaller cav1 currents and robust Ca^2+^ buffering predominantly mediated by calbindin [202,203]. One drawback of the slow Ca^2+^ oscillations in SNpc dopaminergic neurons is the Ca^2+^ entry into the mitochondria, which is needed to sustain ATP production with potential for creating redox imbalance [204,205,206]. 

A second aspect is the functional reserve/resilience of neuronal populations and network compensation under extrenuous demands [134]. In this regard, a common feature of subcortical motor networks is their extensive connectivity and considerable redundancy in the control of motor function [19]. Although we do not have a complete map of human connectome, extrapolation of the known connectivity in the rodent nervous system reveals extensive projections among the brainstem (SNpc, LC and reticular nuclei, including GRN and PAG), striatum, pallidum, thalamus and cortical nuclei, which often are reciprocal [207]. Hence, the following can be argued: (**i**) Networks with high network threshold (i.e., redundancy) are relatively less sensitive to major dysfunction, while networks with low threshold exhibit impaired compensatory response [134]. This implies that comparatively lower loss of neurons would lead to dysfunction in autonomic systems (i.e., due to the LRP affection in autonomic ganglia and dmX/IX) and result in prodromal PD [9,10,142] compared to the estimated 30–50% neuronal loss observed in SNpc prior to the emergence of clinical motor disability [1,2,20]; and (**ii**) the network dysfunction in PD does not necessarily correlate with the trans-synaptic LRP propagation [8,20,134]. For instance, not all projection/reciprocal innervation regions of LC, which are considered the ‘hotspot’ of LRP in early PD, develop robust LRP lesions, e.g., cerebellum and central nucleus of amygdale are relatively spared [20,208].

Lastly, animal studies suggest that neuroinflammatory response of glial cells in response to fibrillar α-syn and/or formation of α-syn inclusion pathology in glial cells [107,108] may also render the local microenvironment conducive to α-syn propagation and neurotoxicity [23,101,169,175]. Human post-mortem studies in synucleinopathies show variable degrees of diffuse α-syn accumulation in the astroglial cells, which is morphologically distinct from the compact neuronal LRP and lacks histological markers of LB pathology (e.g., ubiquitin/p62 immunopositivity) [108]. Cultured astroglia readily internalize extracellular α-syn in vitro, either added exogenously to the culture medium or within the conditioned media from α-syn expressing neuronal cells and in neuron-glia co-culture experiments [108,209]. A substantial number of animal studies also support the view of possible neuron-to-astroglia transmission of aggregated α-syn. For instance, transgenic mice overexpressing human α-syn (wild type or mutant A53T; under the *PDGFβ* or mouse *Prnp* promoters, respectively) in neurons show glial accumulation of α-syn aggregates, which appears at stages when neuronal α-syn inclusion pathology is clearly established [209] or occurs significantly later, implicating neuron-to-astroglia transmission [101,175]. Moreover, inducible expression of the aggregation prone human mutant A53T α-syn in astrocytes (under the astroglial *GFAP* promoter; GFAP-tTA/tetO-α-syn) results in the loss of dopaminergic neurons and neuroinflammation, although it was not clear if glia-to-neuron transmission of aggregated α-syn occurred since no histological data on neuronal α-syn pathology were reported [210].

It is worthwhile to mention that neuronal loss in SNpc is associated with extracellular release of neuromelanin, which is phagocytosed by glial cells and there is evidence for melanin-induced microglial activation in early PD and in the rat SN [180,181,211]. Although this implies that the neuroinflammatory processes are contributors to the disease, some recent reports indicate that activated microglia may also be a source of neurotrophic factors and play a neuroprotective role (Figure 2) [180,212].

## 5. Future Prospects and Opportunities

Apart from the rare forms of familial PD, idiopathic PD runs a protracted course over 15–20 years. With an average number of ~3.6% SNpc neurons affected by LRP, and an estimated lifespan of ~6.2 months in neurons bearing pathological α-syn inclusions before demise, the observed loss of neurons in SNpc at the time of clinical presentation seems to support a relentless course driven by α-syn aggregation in the CNS [192,213]. As more evidence becomes available, i.e., brain imaging, biomarkers in biological fluids or digital tools in clinical practice, it is likely that the two viewpoints discussed above (i.e., Dual-hit hypothesis and Neuronal vulnerability hypothesis) may not be as irreconcilable and could potentially guide a refined understanding of the etiology and symptomatology of PD, especially with respect to the prodromal non-motor symptoms [10]. In this regard, the roles of co-existing neuropathologies and the involvement of white matter in PD are often overlooked [214]. For instance, deposition of tau has been observed in association with LRP, particularly in neurons of LC, basal forebrain and amygdala, and recent studies indicate that tauopathy in PD preferentially affects the nigrostrial neurons than compared with global tauopathy of the Alzheimer type [215,216,217]. Moreover, some clinical features of PD (i.e., rigidity and gait apraxia) in the absence of resting tremor are also seen in rare movement disorders such as progressive supranuclear palsy and corticobasal degeneration [5]. In these disorders, tauopathy affects the basal ganglia and brainstem nuclei, including the SN [5,218]. The akinetic-rigid PD, which is diagnosed in ~50% of patients, preferentially affects the elderly (in contrast to the tremor dominant, which has a younger age of onset), and is frequently associated with cognitive declinesimilar to the age related tauopathies [5,7]. These observations suggest that LRP and tauopathy could engage common pathogenic processes and research in the two fields has the potential to be mutually informative.

As for the prospects in PD research in the near future, we expect significant developments in three areas: (**i**) Novel therapeutic modalities, especially stem cells and viral gene therapy; (**ii**) biomarker discovery, including the use of digital technologies; and (**iii**) refinements in the disease models, ideally towards prodromal PD-like phenotypes. The status of the development of therapeutic modalities and novel candidates for therapy has been reviewed elsewhere [219,220,221,222], hence, we will focus on the latter two aspects. The first is the advancement in the biomarker discovery and technologies that can allow the monitoring of the disease extent (e.g., neuronal loss) and possibly subtle neuronal dysfunction.

Measurements of total or modified forms of α-syn (e.g., p-S129) in biological fluids have been the focus of investigation over the last decade with conflicting results in the levels detected by immunodetection ELISA methods or correlation to the disease severity. Recent studies indicate that the detection of oligomeric and p-S129-α-syn in the cerebrospinal fluid may be promising biomarker candidates in PD, with the ratio of oligomeric/total α-syn greatly improving the sensitivity and specificity of these assays [223,224]. Significant technical improvements have also been made for the detection of protein aggregates in biological fluids that rely on the templated seeding mechanism (e.g., amyloidogenic seeds converting the native protein into misfolded conformers), such as real-time quaking-induced conversion (RT-QuIC) and protein misfolding cyclic amplification (PMCA) [223]. However, to date, these methods are still restricted to research purposes. By comparison, a number of functional neuroimaging approaches, such as the measurement of presynaptic dopamine or dopamine transporter (DAT) and metabolism of L-dopa, has shown to differentiate PD from controls with impressive specificity and sensitivity [225,226]. An easily applicable and low-cost neuroimaging approach using transcranial sonography also distinguishes the pathological affection of SN, i.e., abnormal extension of SN echogenicity, in the majority of patients [227]. Similarly, myocardial scintigraphy (using 123I-metaiodobenzylguanidine) has also been successfully used to detect cardiac sympathetic denervation in early PD and in the differential diagnosis [228]. Another exciting development is in the area of digital/telemetric technologies, which can serve as an aid in detecting early neurological dysfunction, guide in patient monitoring and response to therapy [229,230]. Although, more scientific data and rigorous analyses on their utility are still missing, these can be useful clinical tools in the detection of sleep disorders, gastrointestinal problems (i.e., dysphagia, salivation, constipation) and tremor.

Lastly, there is a dire need for refinements in experimental models that can be used to study motor deficits largely due to the extranigral α-syn pathology and non-motor symptoms, such as postural instability and pain [9,10]. Several animal models, based on transgenic α-syn overexpression or viral mediated α-syn somatic gene transfer, recapitulate aspects of PD-like α-syn pathology, such as loss of dopamine and motor phenotypes due to basal ganglia dysfunction [23,167,169]. Among the transgenic models, the mice expressing human wild type α-syn under the *Thy1* promoter have been consistently reported to exhibit non-motor phenotypes that are relevant to PD, such as cognitive impairment, olfactory dysfunction, constipation and changes in the circadian rhythm [231,232]. Given the nigro-centric neuropathological context of PD, it is understandable that the animal models have largely focused on the nigrostriatal dysfunction. However, it would be interesting to study sensorimotor phenotypes by selective α-syn lesions in extra-nigral locations, such as LC, GRN, PAG and the hypothalamus. In this regard, two recent studies show that viral mediated induction of mutant A53T α-syn in LC [233] or transgenic overexpression of human wild-type α-syn under control of the noradrenergic-specific dopamine β-hydroxylase promoter [234] results in the development of PD-like α-syn pathology, neuroinflammation and behavioral deficits in the latter [234]. 

The GRN is part of the brainstem reticular nuclei, which, according to Braak staging, is also affected very early in the disease [18,133]. The neuronal populations in GRN are extensively connected to several brain regions including LC and cerebellar nuclei and via their descending projections to spinal motor systems (i.e., premotor and motor neurons) serve as the ‘gain-setting mechanism’ in the control of movement and posture [19]. In a PFF model of synucleinopathy (M83 transgenic line expressing the human mutant A53T α-syn) [235] several laboratories, including our own, have shown that, after the initial appearance of α-syn pathology (p-S129) in the spinal cord following intramuscular PFF inoculation, periventricular regions of brainstem (i.e., medullary reticular nuclei, LC, pontine GRN and midbrain PAG) are affected long before the emergence of movement disability [170,175,236]. This ‘prodromal’ phase coincides with the emergence of a sensorimotor deficit exhibited as mild degree of hindlimb clasping [102,236], which is a behavior observed in rodents with lesions in basal ganglia and cerebellum [237]. Moreover, the (intramuscularly) PFF inoculated M83 mice exhibit a hunched posture at the end-stage, although it is not clear if this phenotype is due to neuronal dysfunction in higher the brain region or if it results from the significant loss of spinal motor neurons [101]. It is worthwhile to indicate that the PFF based models have several limitations to qualify as bona fide PD models, including the lack of both the dopaminergic cell loss and significant α-syn pathology in SN [23,169,236]. Nevertheless, several groups have demonstrated that these models exhibit α-syn pathology beyond the site of PFF inoculation (i.e., intracerebral or peripheral) and putative ‘trans-synaptic’ spreading [23]. 

The neuronal populations in PAG are also heterogeneous and have several projections that link forebrain structures to the reticular nuclei of the brainstem [238]. This phylogenetically ancient region has been implicated in autonomic regulation (possibly via hypothalamus), circadian rhythm and pain modulation (via descending projections to the raphe magnus and spinal nociceptor neurons) [238,239]. In the PFF inoculated (via the intramuscular route) M83 transgenic mice, abundant α-syn pathology (p- S129 α-syn) in the PAG (and spinal cord) was associated with mechanical allodynia and impaired pain response [102]. However, we are still in the preliminary stages in terms of inferring whether the impaired pain perception was due to central neuronal/nociceptive dysfunction or the loss of normal nerve function as measured by nerve conduction velocity and myelin damage in the nerve dorsal roots [102]. Hence, quasi-selective induction of α-syn aggregation in central pain processing centers, i.e., PAG, may unravel relevant mechanisms for pain, which is reported in a considerable number of PD patients [10].

## 6. Conclusions

Since the early discovery of α-syn as a major component of LB pathology in PD, and genetic linkage between mutations in the *SNCA* with rare forms of familial PD [30,240,241,242], significant progress has been made to make a compelling case for a pathogenic role of α-syn aggregation in PD and related diseases [12,17,240]. However, there are several aspects that represent the missing links between α-syn aggregation in the CNS and the onset and/or progression of clinical PD symptomatology. This is illustrated by the fact that despite a growing consensus on the putative downstream mechanisms of α-syn neurotoxicity following its misfolding and/or aggregation [15,17], the identity of causative factor(s) that promote the initial pathological conversion of α-syn into neurotoxic species in PD is largely unsettled. Apart from the case of rare familial forms due to specific genetic mutations in the *SNCA* locus (i.e., increased protein expression due the gene dosage effect and tendency to form oligomers as a result of certain point mutations), the nature of mechanism(s) that promote α-syn aggregation in other forms of PD (genetic or idiopathic) remains elusive, and the mechanisms are likely to be of multi-factorial origin [1]. For instance, a sizeable proportion (8–14%) of autopsy proven PD cases reveal mutations in the gene encoding glucocerebrosidase (*GBA*) associated with perturbed lysosomal function [243] and potentially favor α-syn aggregation as a result of ensuing lipid accumulation and defective autophagy [15,243]. A pathogenic relevance of defective autophagy promoting α-syn aggregation in PD is also supported by the studies in cellular and animal models overexpressing PD-associated mutations in the leucine-rich repeat serine/threonine-protein kinase 2 (LRRK2) [244], vacuolar protein sorting-associated protein 35 (VPS35) [245] and showing putative interactions of parkin with mutant glucocerebrosidase [246]. Thus, it could be argued that rectifying the pathological decrease in autophagic flux may hold promise in mitigating the neurotoxic effects of aggregated α-syn in at least a subset of PD cases. Mechanistic studies in different PD populations using refined approaches, e.g., advanced genetic studies in patient derived induced pluripotent stem cells [247], will potentially reveal whether the defects in autophagic flux of lipids and/or proteins are a generalized feature in PD or if additional contributing mechanisms underlie the etiology of pathological α-syn accumulation in different PD populations.

Another relevant consideration is the establishment of a framework for the identification of patient specific factors, such as co-existing neuropathology (e.g., tau [218]) and co-morbidities that predispose to age related neurological dysfunction (e.g., impaired glycemic control) that may inform on the clinical phenotypes in relation to the distribution and/or progression of α-syn pathology [248,249]. For instance, while tau pathology is a pronounced post-mortem feature in the cases of familial PD due to LRRK2 mutations, not all PD cohorts with pathogenic LRRK2 mutations exhibit α-syn LRP [250,251]. It is worth considering that the paucity of LRP (inclusions containing fibrillar α-syn, which is ubiquinated [114]) in these subsets of PD cases does not rule out the existence of oligomeric α-syn in the brain, since α-syn oligomers have been reported in the cerebrospinal fluid of individuals who carry LRRK2 mutations, either with a PD diagnosis [252] or in neurologically normal volunteers [253]. However, research reagents (e.g., conformational antibodies) that can detect oligomeric α-syn have not been systematically studied to the extent that they are generally accepted in their own accord as the tissue biomarkers of pathological α-syn accumulation. Moreover, the specificity of these reagents to unambiguously recognize ‘oligomeric’ α-syn conformations without binding to fibrillar α-syn has also been contested [254].

In conclusion, the wealth of information about PD symptomatology, extensive characterization of the neuropathological findings and refinements in animal models hold promise for meaningful discoveries that may yield potential biomarkers of disease as well as guide the development of disease modifying therapies.

## Data Availability

All the data cited in the article can be accessed in original research studies provided in the bibliography under the references.
